# Bovine dairy complex lipids improve *in vitro* measures of small intestinal epithelial barrier integrity

**DOI:** 10.1371/journal.pone.0190839

**Published:** 2018-01-05

**Authors:** Rachel C. Anderson, Alastair K. H. MacGibbon, Neill Haggarty, Kelly M. Armstrong, Nicole C. Roy

**Affiliations:** 1 Food Nutrition & Health Team, Food & Bio-based Products Group, AgResearch Grasslands, Palmerston North, New Zealand; 2 Riddet Centre of Research Excellence, Massey University, Palmerston North, New Zealand; 3 Fonterra Research and Development Centre, Palmerston North, New Zealand; Universitatsklinikum Hamburg-Eppendorf, GERMANY

## Abstract

Appropriate intestinal barrier maturation is essential for absorbing nutrients and preventing pathogens and toxins from entering the body. Compared to breast-fed infants, formula-fed infants are more susceptible to barrier dysfunction-associated illnesses. In infant formula dairy lipids are usually replaced with plant lipids. We hypothesised that dairy complex lipids improve *in vitro* intestinal epithelial barrier integrity. We tested milkfat high in conjugated linoleic acid, beta serum (SureStart™Lipid100), beta serum concentrate (BSC) and a ganglioside-rich fraction (G600). Using Caco-2 cells as a model of the human small intestinal epithelium, we analysed the effects of the ingredients on trans-epithelial electrical resistance (TEER), mannitol flux, and tight junction protein co-localisation. BSC induced a dose-dependent improvement in TEER across unchallenged cell layers, maintained the co-localisation of tight junction proteins in TNFα-challenged cells with increased permeability, and mitigated the TEER-reducing effects of lipopolysaccharide (LPS). G600 also increased TEER across healthy and LPS-challenged cells, but it did not alter the co-location of tight junction proteins in TNFα-challenged cells. SureStart™Lipid100 had similar TEER-increasing effects to BSC when added at twice the concentration (similar lipid concentration). Ultimately, this research aims to contribute to the development of infant formulas supplemented with dairy complex lipids that support infant intestinal barrier maturation.

## Introduction

The human intestine has two roles that are seemingly conflicting; it must enable the absorption of nutrients while also preventing the entry of potentially disease-causing components into the body. This barrier function takes around two years to fully develop, from the initial gut closure which develops during the first week of life followed by the development of the intestinal immune system and microbial colonisation which take up to 2 years to complete (reviewed in [1]). The complex processes involved in this maturation are modulated by human breast milk, which is reflected by differences in intestinal physiology between breast-fed versus formula-fed infants in relation to barrier integrity, absorption capacity, and microbiota composition [2]. The intestinal barrier of formula-fed infants is less resilient to challenges, and as a result they are more susceptible to conditions such as infectious diarrhoea, necrotising enterocolitis, and allergic gastroenteropathy [3].

Appropriate intestinal barrier maturation is also essential for life-long health. Barrier dysfunction in adulthood is a critical factor in predisposition to intestinal diseases [4] and is also associated with autoimmune diseases in other parts of the body [5]. These illnesses are often linked with increased intestinal permeability (often called ‘leaky gut’) and are more common in adults that were formula-fed compared to those that were breast-fed [6] indicating that sub-optimal intestinal barrier maturation in early-life may have long-term consequences.

One difference between human milk and infant formula that may contribute to these differences in infant intestinal epithelial barrier maturation is the lipid composition [7]. Typically in infant formulas the dairy lipids are replaced with lipids from plant sources. This has the consequence of reducing the levels of beneficial complex lipids in infant formula. For example, phospholipids and sphingolipids (e.g. gangliosides), which are known to play a role in infant intestinal maturation [8,9], are present in mammalian milks but not plant-derived lipid fractions.

Our over-arching aim is to determine whether bovine dairy lipid fractions support appropriate intestinal barrier maturation and resilience by increasing the integrity of the intracellular tight junctions that control permeability. As a first step, we tested hypothesis that dairy lipid fractions improve *in vitro* epithelial tight junction integrity. We selected a number of lipid fractions with differing compositions which are summarised in Table 1. This included: 1) High CLA milkfat which contains 5% CLA compared to 1.2% in standard milkfat; 2) Beta serum (SureStart^TM^Lipid100) which is a by-product from manufacturing of anhydrous milk fat and is rich in milkfat globule membranes and phospholipids; 3) Beta serum concentrate (BSC) which is a low lactose version of beta serum (6.6 versus 42% lactose); and 4) Ganglioside-rich fraction (G600) which is a protein-free fraction containing higher concentrations of gangliosides and phospholipids than SureStart^TM^Lipid100 and BSC.

**Table 1:** Composition of the bovine dairy lipid fractions used in these experiments.

Milk components (% total)		Standard milkfat		High CLA milkfat		Beta serum (SureStart^TM^Lipid100)		Beta serum concentrate (BSC)		Ganglioside-rich fraction (G600)
Lactose		-		-		42		6.6		56
Total protein		-		-		30		52		-
Membrane-bound protein		-		-		7.6		13.2		-
Total fat		99.9		99.9		20.24		36.2		30.5
Triacylglycerols		99.9		99.9		12.3		22.5		15.2
Conjugated linoleic acids		1.17		5.04		0.22		0.4		0.44
Phospholipids		-		-		7.9		13.7		15.3
Glycerophospholipids		-		-		6.2		10.6		13.0
Sphingomyelin		-		-		1.7		3.1		2.3
Gangliosides		-		-		0.33		0.63		1.43
Ash		-		-		6.6		5.2		7.2

To mimic the small intestinal epithelium we used the Caco-2 cell line. Although these cells originated from the colon morphologically and functionally they are characteristic of small intestinal enterocytes [10]. Using this cell line we analysed the effect of the diary lipid fractions on *in vitro* measures of intestinal barrier integrity, including trans-epithelial electrical resistance (TEER), mannitol flux, and confocal microscopy of key intracellular tight junction proteins. The effects of the lipid fractions on both unchallenged cells and those challenged with permeability-inducing compounds tumour necrosis factor alpha (TNFα) and lipopolysaccharide (LPS) were examined.

## Results

### BSC and G600 increased TEER across unchallenged but not TNFα-challenged cell monolayers

**Fig 1:**
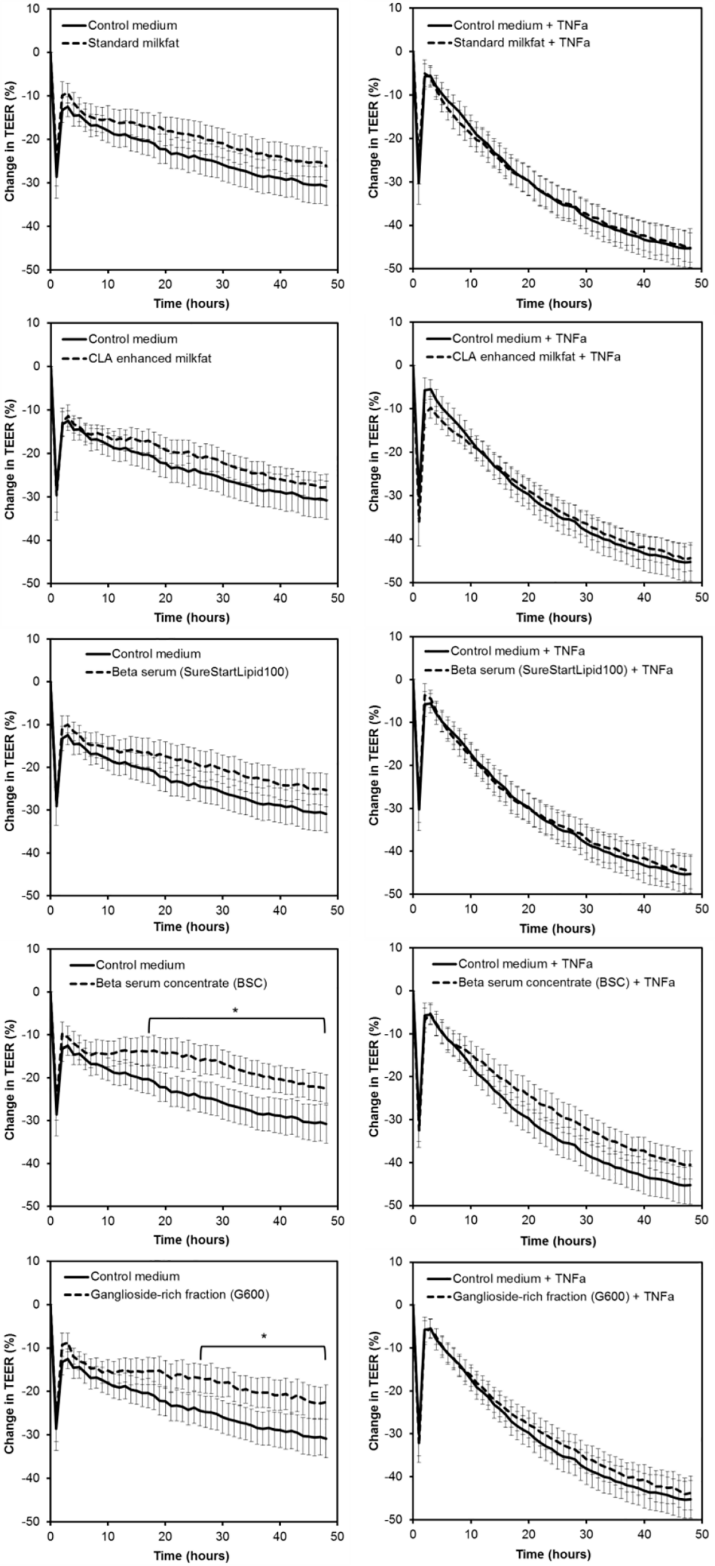
Effect of dairy lipid fractions on the trans-epithelial electrical resistance (TEER) across healthy or TNFα-challenged Caco-2 cell monolayers. All lipid fractions were suspended at 1 mg/mL in control medium. The data from three independent assays each with four replicates per treatment were combined (total n = 12 per treatment). Values shown are the means and the error bars show the SEM. * Difference between the lipid treatment and control medium was greater than the 5% LSD (7.6) over the time period indicated.

The effects of the dairy lipid fractions on TEER across unchallenged and TNFα-challenged Caco-2 cell monolayers are shown in Fig 1. The Caco-2 cell monolayers treated with control medium alone and with control medium with TNFα behaved as expected. In both cases there was an initial drop in TEER due to the cell layers being disturbed by the addition of the treatment solutions, which recovered by 2 hours. In the control medium group the TEER decreased slowly over time as the nutrients in the medium were depleted. In the TNFα group the TEER decreased more rapidly, and there was a difference in TEER between control medium and TNFα-challenged cells from 19 hours onwards (P<0.05).

For the unchallenged cell monolayers (no TNFα treatment), the *treatment x time* effect was significant (P<0.001) so further comparison between treatment groups was warranted. Standard milkfat, high CLA milkfat and beta serum did not alter the TEER compared to control medium at any time point. However, the enriched fractions, BSC and G600, caused a sustained increase in TEER compared to control medium (Fig 1). For the TNFα-challenged cell monolayers, the *treatment x time* effect and the *treatment* effect were not significant (P = 0.639 and P = 0.289, respectively). Therefore, none of the dairy lipid fractions overcame the decrease in TEER induced by TNFα.

### BSC and ganglioside-rich fraction G600 did not alter mannitol flux

**Fig 2:**
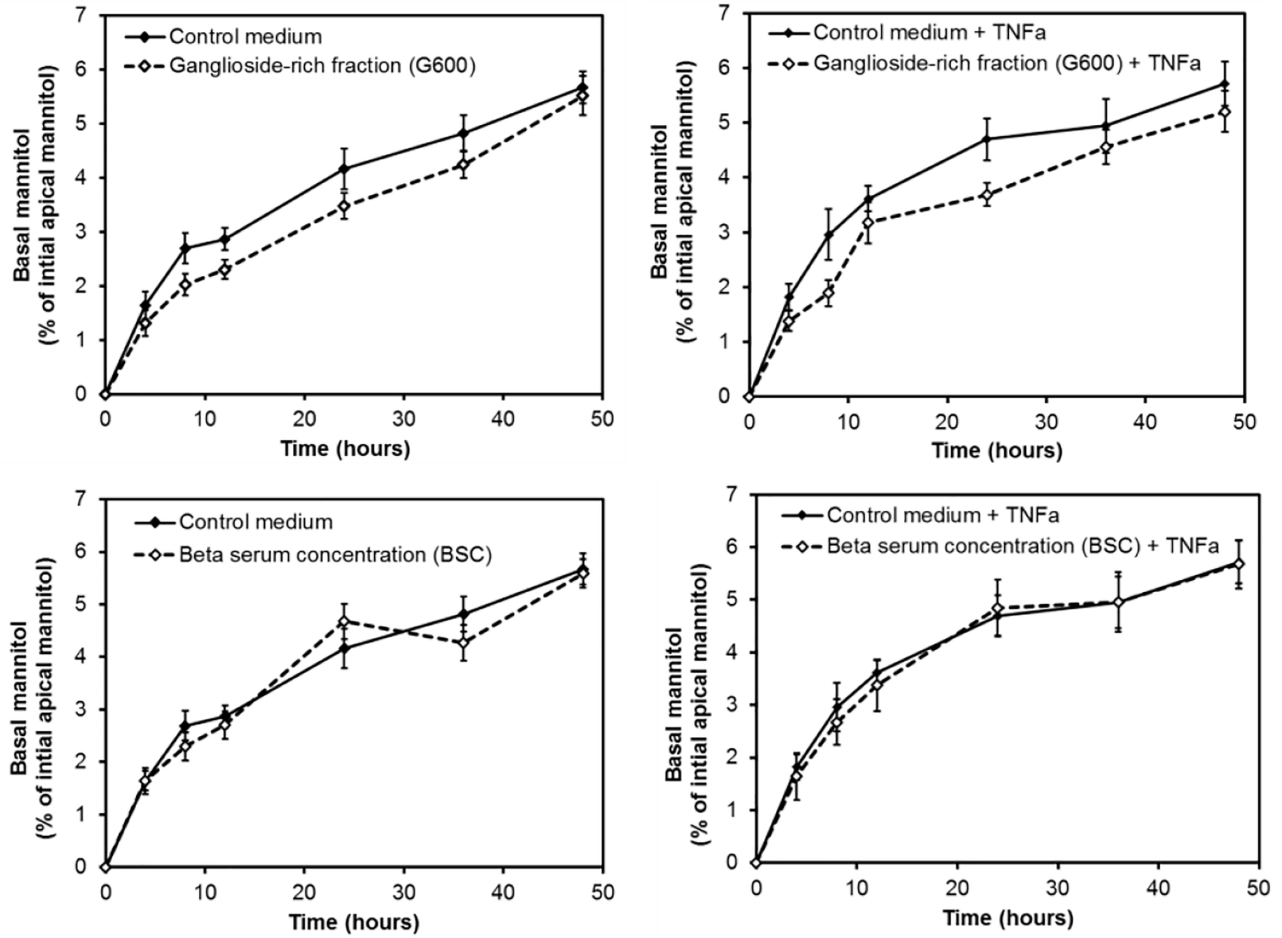
Effect of dairy lipid fractions on the flux of mannitol across unchallenged or TNFα- challenged Caco-2 cell monolayers. Both lipid fractions were suspended at 1 mg/mL in control medium. The data from three independent assays each with four replicates per treatment were combined (total n = 12 per treatment). Values shown are the means and the error bars show the SEM.

The two dairy lipid fractions that increased TEER across healthy Caco-2 cell monolayers were tested in the mannitol flux assay as shown in Fig 2. As expected, the amount of mannitol that had passed across the cell layers to the basal compartment increased over time for all treatments. The analysis indicated that there was no significant *treatment x time* or *treatment* effect (P = 0.346 and P = 0.146, respectively). Therefore, neither dairy lipid fractions altered the flux of mannitol over time.

### BSC maintained ZO-1 and occludin co-localisation during TNFα challenge, but G600 did not

**Fig 3:**
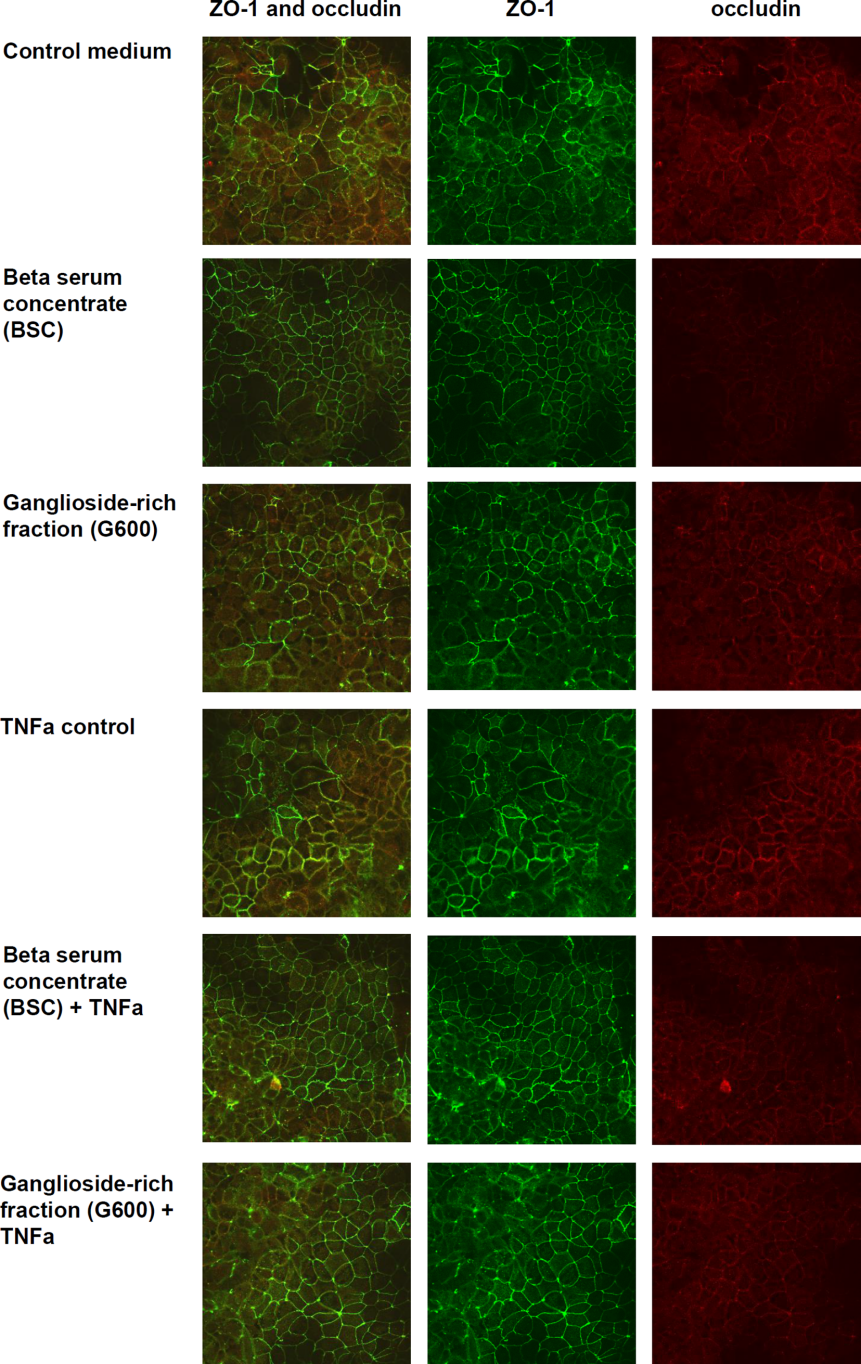
Effect of dairy lipid fractions on the intensity of Caco-2 cell fluorescently stained ZO-1 and occludin proteins. Both lipid fractions were suspended at 1 mg/mL in control medium and the treatment period was 48 hours. The images shown are typical of 9 images taken per treatment group (3 images from each of 3 membranes) at a magnification of 60x.

**Fig 4:**
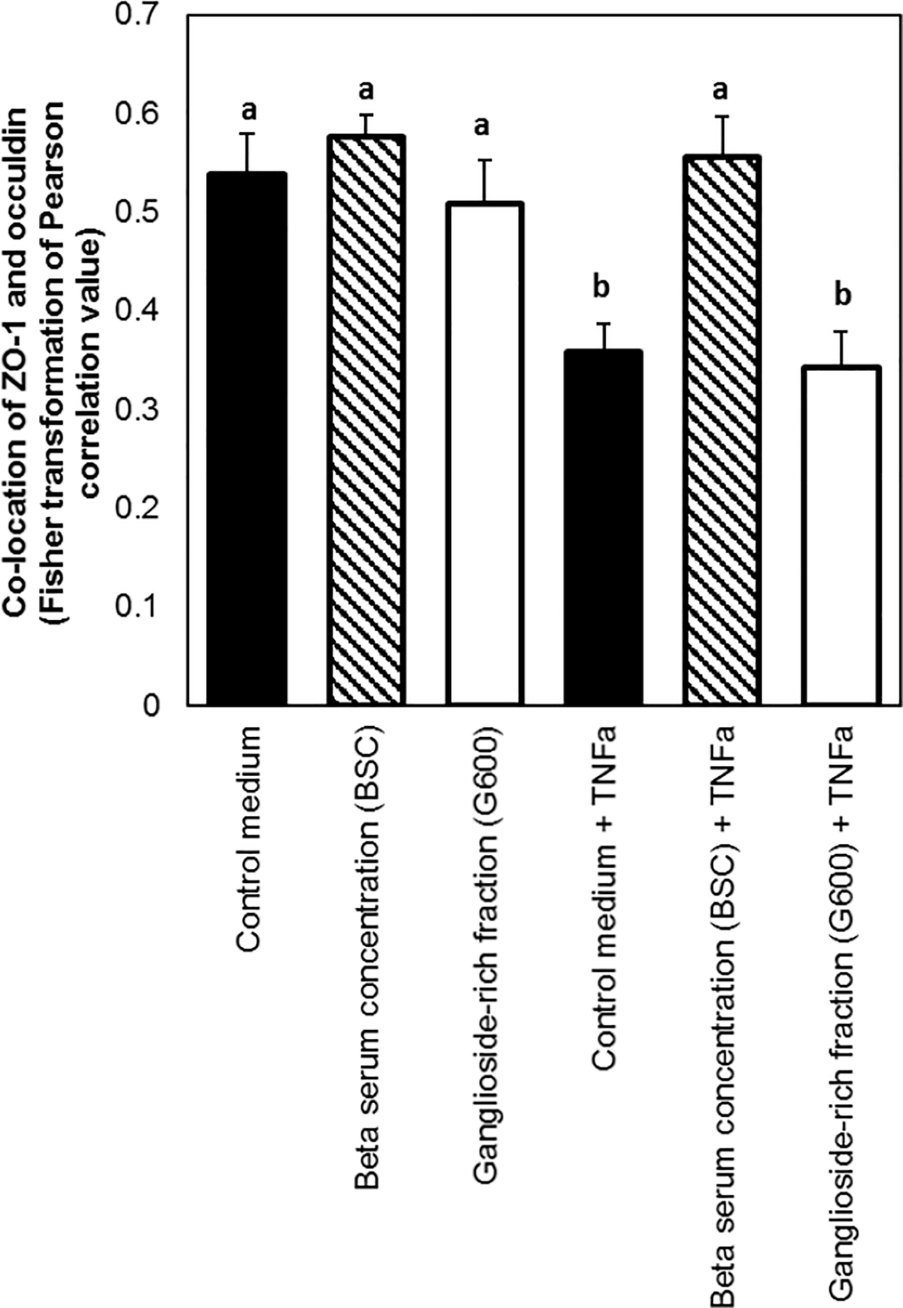
Effect of dairy lipid fractions on the co-localisation of ZO-1 and occludin in Caco-2 cell monolayers. Both lipid fractions were suspended at 1 mg/mL in control medium and the treatment period was 48 hours. Values shown are the means and the error bars show the SEM. The lowercase letters indicate treatments that were significantly different according to the Tukey’s 95% confidence intervals test.

The effect of the two TEER-increasing dairy lipid fractions on the localisation of tight junction proteins was investigated using confocal microscopy. Typical images for each treatment group are shown in Fig 3. The mean Pearson’s correlation values for co-location of ZO-1 and occludin were either weak (0.3–0.5) or moderate (0.5–0.7), with no treatments resulting in a strong correlation. The *treatment* effect was significant (P<0.05) so the pair-wise analysis was carried out. As shown in Fig 4, there was no difference between the cells treated with control medium and the dairy lipid fractions in the absence of TNFα. As expected, there was lower co-localisation of the tight junction proteins in the cells treated with TNFα compared to those untreated. This was mitigated by BSC, which caused co-localisation similar to control medium, but not by G600.

### BSC had a dose-dependent effect of TEER but SureStart^TM^Lipid100 did not

**Fig 5:**
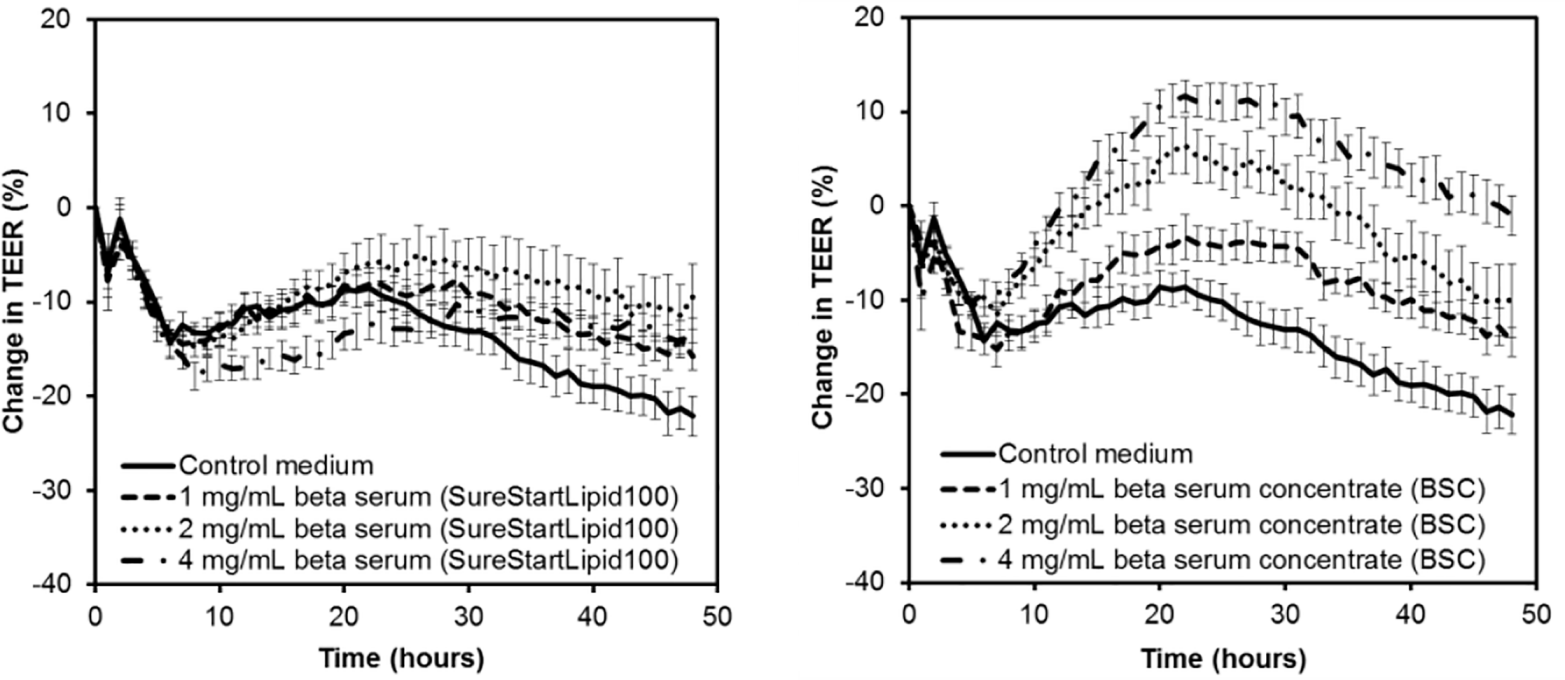
Effect of doses of low lactose beta serum and beta serum on the trans-epithelial electrical resistance (TEER) across healthy Caco-2 cell monolayers. The data from three independent assays each with four replicates per treatment were combined (total n = 12 per treatment). Values shown are the means and the error bars show the SEM. The 5% LSD between treatment groups at a given time point is 5.96.

SureStart^TM^Lipid100 contains around half of the lipids in BSC, so it was hypothesised that twice the concentration of SureStart^TM^Lipid100 would have a similar effect on TEER as BSC concentrate. The effect of dose on the ability of SureStart^TM^Lipid100 and BSC to alter TEER is shown in Fig 5. The *treatment x time* effect was significant (P<0.001) so comparison between treatment groups was performed.

BSC had a dose-dependent effect on TEER, with higher concentrations causing greater increases in TEER over longer time frames; 2 mg/mL was higher than 1 mg/mg over 10 to 37 hours, and 4 mg/mL was higher than 2 mg/mL over 24 to 48 hours. This dose-dependent effect was not apparent for SureStart^TM^Lipid100. In agreement with the hypothesis, for 2 mg/mL SureStart^TM^Lipid100 and 1 mg/mL BSC, which contained similar amounts of lipids, where there was no difference between the treatments at any time point. However, at the higher concentrations of 4 mg/mL SureStart^TM^Lipid100 and 2 mg/mL BSC, BSC induced higher TEER from 42 hours.

### SureStart^TM^Lipid100, beta serum concentrate (BSC) and G600 increased TEER across LPS-treated cell monolayers

**Fig 6:**
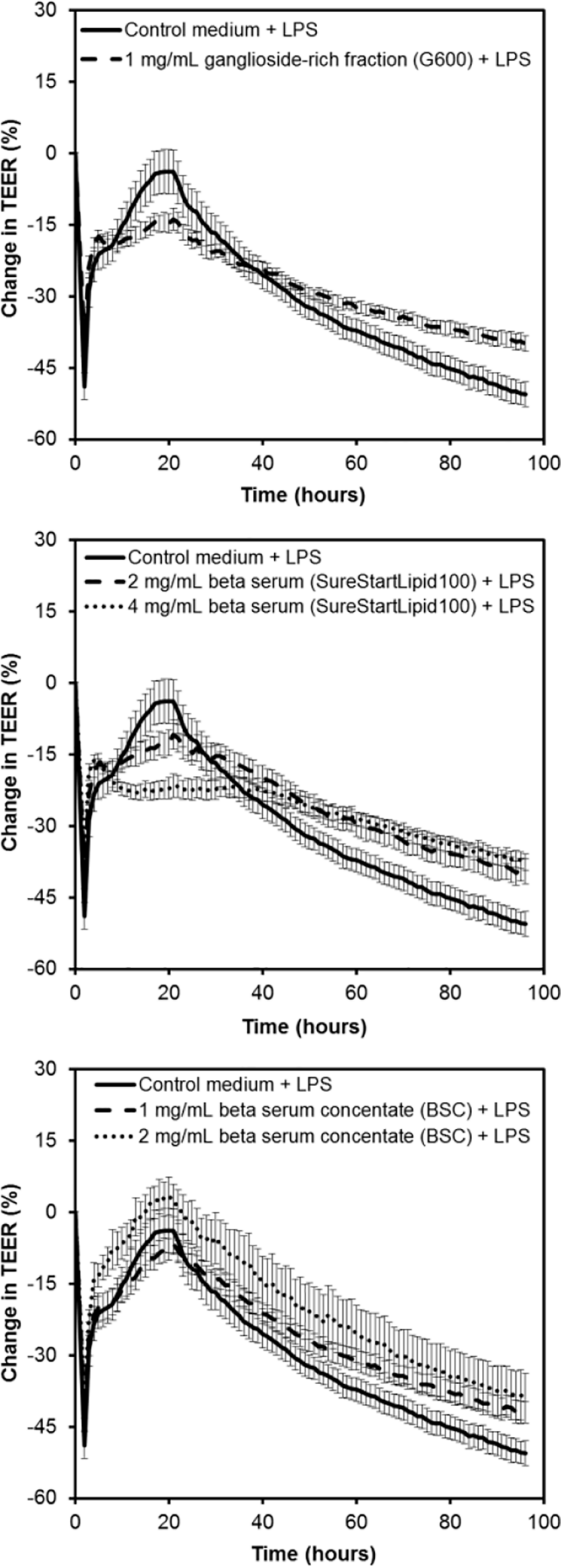
Effect of dairy lipid fractions on the trans-epithelial electrical resistance (TEER) LPS-treated Caco-2 cell monolayers. The data from three independent assays each with four replicates per treatment were combined (total n = 12 per treatment). Values shown are the means and the error bars show the SEM. The 5% LSD between treatment groups at a given time point is 3.21.

The ability of the dairy lipid fractions to overcome the negative effect on TEER caused by LPS was investigated. The effect of the dairy lipids fractions of interest on the TEER across cell monolayers challenged with LPS is shown in Fig 6. Treatment with LPS initially caused the TEER to increase, however over time the TEER decreased indicating that LPS had a detrimental effect on TEER as expected. The *treatment x time* effect was significant (P<0.001) so comparison between treatment groups was completed.

Initially cell monolayers treated with G600 and LPS had a lower TEER than those treated with LPS alone (11 to 30 hours). However, later in the experiment when the LPS-induced reduction in TEER was apparent, monolayers treated with G600 had a higher TEER (52 to 96 hours). The effect of BSC on TEER across LPS-treated cell monolayers was dose-dependent, with 2 mg/mL inducing a higher TEER than 1 mg/mL for the majority of the experiment (3 to 95 hours). From 12 to 41 hours, 1 mg/mL BSC caused a higher TEER than 1 mg/mL G600, indicating that higher ganglioside concentration did not correlate with increased TEER.

In contrast to the results with BSC, the TEER increasing effect of SureStart^TM^Lipid100 was not dose-dependent, with 2 mg/mL and 4 mg/mL causing similar effects from 39 hours onwards. Like the results with healthy cells, 2 mg/mL SureStart^TM^Lipid100 and 1 mg/mL BSC, which contained similar concentrations of lipids, had comparable effects. However, treatment with 4 mg/mL SureStart^TM^Lipid100 resulted in a lower TEER than treatment with 2 mg/mL BSC.

## Discussion

In agreement with our hypothesis, bovine dairy lipid fractions improved *in vitro* indicators of small intestinal epithelial barrier integrity. This beneficial effect was apparent both in healthy cells indicating they may improve infant intestinal maturation, and in LPS-challenged cells suggesting they may increase the resilience of the intestinal epithelium to infections.

Of the treatments tested BSC had the greatest effects. It induced a dose-dependent increase in TEER across healthy cell layers, maintained the co-localisation of ZO-1 and occludin in TNFα-challenged cells, and reduced the decrease in TEER caused by LPS. The ganglioside-rich fraction, G600, also increase TEER across healthy and LPS-treated cells but it did not alter the co-location of ZO-1 and occludin in TNFα-challenged cells.

SureStart^TM^Lipid100 had similar TEER increasing effects across healthy and LPS-treated cells as BSC when added at twice the concentration (similar lipid concentration). However, unlike BSC, the TEER-enhancing properties of SureStart^TM^Lipid100 did not increase with increasing dose. Given that the difference between SureStart^TM^Lipid100 and BSC is that BSC has the majority of the lactose removed, this suggests that the lactose present in SureStart^TM^Lipid100 may be masking the effects of the lipids at higher concentrations.

The improvement in measures of barrier integrity in response to the dairy lipid fractions could be caused by the gangliosides present. Gangliosides have been shown to mitigate against the degradation of occludin in rats challenged with LPS [11]. Mechanisms such as the gangliosides’ ability to reduce the adhesion of pathogenic bacteria [12] and promote the growth of infant gut-associated bifidobacteria [13] have been proposed. However, there were no bacteria present in our experimental setup, indicating that either gangliosides also act directly on the epithelial cells or that another compound is responsible for the beneficial effect.

The second possibility may be more plausible because G600, which has more than twice the amount of gangliosides than BSC, did not increase TEER more than BSC. However, G600 also contains a large amount of lactose (56%), which appears to impair the barrier integrity in the assay evident by the discrepancy in the dose response of high-lactose SureStart^TM^Lipid100 versus low-lactose BSC. To fully understand this, experiments with ganglioside-rich fraction with lactose removed or pure ganglioside are required.

Alternatively, the beneficial effects could be due to sphingomyelin which is most abundant in BSC compared to the other lipid fractions. Sphingomyelin is both a phospholipid (has a phosphate group) and a sphingolipid (has a sphingosine group). Although milk phospholipids are known to play an important role in infant intestinal maturation [8], their effects on TEER and tight junction composition have not been reported. However, sphingosine-1-phosphate, a sphingolipid produced by intestinal epithelial cells, is known to enhance TEER [14]; therefore, it is plausible that dietary sphingolipids, such as sphingomyelin, could have similar effects.

Milkfat high in CLA did not improve TEER across healthy or TNFα-challenged cell layers. Pure CLA at a concentration of 14 μg/mL was previously shown to increase TEER development when used throughout Caco-2 cell growth and differentiation (0 to 21 days) compared to standard medium [15], but this improvement in TEER was not apparent when high CLA milkfat, with CLA concentration of 50 μg/mL, was added to already differentiated Caco-2 cell layers here.

Seemingly incongruously, neither of the dairy lipid fractions that improved TEER reduced the flux of mannitol across the cell layers. Although TEER and mannitol flux are both measures of paracellular permeability, the TEER assay specifically measures ion permeability, whereas the mannitol flux assay measures the passage of uncharged molecules. Tight junctions are highly dynamic structures, involving more than 50 known proteins, and the independent regulation of TEER and mannitol flux by different tight junction proteins is a known phenomenon [16]. For example, over-expression of occludin results in increased TEER (reduced ion permeability) and increased mannitol flux (increased small molecule permeability), implying that occludin allows selective paracellular flux of uncharged molecules in the presence of electrically sealed tight junctions [16]. In the case of the dairy lipid fractions tested here, they appear to reduce ion permeability but not alter small molecule permeability.

Another potential inconsistency in the results is that fact that the dairy lipid fractions were able to partially mitigate against the TEER decrease caused by LPS but not that caused by TNFα. Both of these pro-inflammatory molecules act via the nuclear factor kappa B (NF-κB) pathway, but the resulting cytokine secretion profiles are different [17], so it is possible that the mechanisms involved are distinct. Alternatively, the differences in effects could be due to the severity of the challenge; TNFα reduced TEER by 40 to 50% after 48 hours of treatment, whereas LPS took 72 hours to reduce TEER to this same extent. The diary lipid fractions may have been able to mitigate against the less serve LPS challenge but not the more severe TNFαchallenge.

The next step in this research will be to determine whether the *in vitro* effects translate into *in vivo* benefits. While *in vitro* models provide useful insights, they are limited in that they do not include the complexity of gastrointestinal system, such as digestion enzymes, resident microbiota and motility, which may alter the effects of the dietary treatments. Therefore, we intend to verify our results using a piglet model because they have gut anatomy and physiology that is similar to humans [18,19]. Like infants, piglets have altered small intestinal morphology and permeability depending on the milk diet composition [20–22], including changes in tight junction structures, making them a suitable model for such studies.

In conclusion, our research showed that dairy lipid fractions, SureStart^TM^Lipid100, BSC, and G600 increase *in vitro* measures of small intestinal barrier integrity both in healthy cells and in those with a low level inflammatory challenge. Ultimately, this research aims to contribute to the development of infant formulas supplemented with bovine dairy complex lipids that support appropriate intestinal barrier maturation and resilience.

## Methods

### Bovine dairy lipid fractions

The bovine dairy lipid fractions summarised in Table 1 were supplied as powders by Fonterra Research and Development Centre (Palmerston North, New Zealand). On the day of the experiments, the dairy lipid fractions were suspended at the required concentration in the appropriate cell culture medium and warmed to 37°C prior to addition to the cell culture assays described below. The researchers were blinded to the composition of the dairy lipid fractions until after the analysis was completed.

### Trans-epithelial electrical resistance assay

To determine whether the bovine dairy lipid fractions affected intestinal barrier integrity, the TEER across an *in vitro* Caco-2 cell monolayer was measured in response to the treatments. The ability of the bovine dairy lipid fractions to overcome the decrease in TEER caused by the pro-inflammatory cytokine TNFα and lipopolysaccharide LPS was also investigated. Authenticated Caco-2 cells (American Type Culture Collection) were grown on Transwell inserts (6.5 mm, polyester, 0.4 μm pore size; Corning Incorporated, Carlsbad, CA) in Medium 199 (M199; Gibco, Invitrogen Corporation, Carlsbad, CA) supplemented with gamma-irradiated triple-filtered 10% foetal bovine serum (FBS; Gibco, Invitrogen Corporation, Carlsbad, CA), 1% non-essential amino acids (NEAA; MEM non-essential amino acids 100x solution; Sigma-Aldrich, St. Louis, MO) and 1% penicillin-streptomycin (Pen-Strep: 10,000 units/mL penicillin G sodium salt and 10,000 μg/mL streptomycin sulphate in 0.85% saline; Gibco, Invitrogen Corporation, Carlsbad, CA) at 37°C in 5% CO_2_ until differentiated as previously described[23]. The day prior to the TEER assay, Transwells were transferred into cellZscope cell modules (nanoAnalytics GmbH, Munster, Germany) and the baseline resistances across the cell layers were measured hourly for 24 hours using the cellZscope computer-controlled system. Caco-2 monolayers that had baseline TEER greater than 400 ohms/cm^2^ were used for assays. At the start of the assay, the apical medium from the Transwells was replaced with the treatment solutions: control medium (M199 supplemented with 1% NEAA and 1% Pen-Strep) or bovine dairy lipid fractions in control medium. FBS was not included in the apical medium in case it interfered with the bioactivity of the lipids. The basal medium was replaced with fresh medium (M199 supplemented with 10% FBS, 1% NEAA, and 1% Pen-Strep), or with a challenge of 100 ng/mL TNFα (Sigma Aldrich, St. Louis, MO) or LPS from *Escherichia coli* 0127:B8 (Sigma-Aldrich, St. Louis, MO) in medium. TEER was measured every hour for 48 hours and the percentage change in TEER at each time point compared to the initial TEER was calculated. Three assay runs each with four replicates per treatment were carried out (total n = 12 per treatment).

### Mannitol flux assay

As an alternative measure of barrier integrity the mannitol flux assay was carried out. TEER is a measure of paracellular ion permeability whereas mannitol flux is a measure of paracellular small molecule permeability. Caco-2 cells were grown on Transwell inserts until differentiated as described for the TEER assay. Caco-2 monolayers that had background TEER greater than 400 ohms/cm^2^ were used for assays. At the start of the assay, the apical medium from the Transwells was replaced with the treatment solutions: control medium (M199 supplemented with 1% NEAA and 1% Pen-Strep) or bovine dairy lipid fractions in control medium. The basal medium was replaced with fresh medium (M199 supplemented with 10% FBS, 1% NEAA, and 1% Pen-Strep), or with a challenge of 100 ng/mL TNFα. Three independent assays each with four replicates per treatment were carried out (total n = 12 per treatment). To each apical insert, 20 μL of 2.5 μCi/mL ^3^H-mannitol (Mannitol, D–[1-3H(N)]; American Radiolabelled Company (ARC), St. Louis, MO) was added at time zero. Every 2 hours 25 μL of basal solution was removed. The samples were mixed with 25 μL Starscint Scintillation Fluid (PerkinElmer, Waltham, MA) and the ^3^H-mannitol was measured using a Luminescence counter (1450 MicroBeta TriLux; PerkinElmer, Waltham, MA). The percentage of ^3^H-mannitol that crossed the cell layer was calculated and plotted against time.

### Confocal microscopy

Caco-2 cells were grown on Transwell inserts until differentiated as described for the TEER assay. At the start of the assay, the apical medium was replaced with the treatment solutions: control medium (M199 supplemented with 1% NEAA and 1% Pen-Strep) or 1 mg/mL dairy lipid fractions in control medium. The basal medium was replaced with fresh medium (M199 supplemented with 10% FBS, 1% NEAA, and 1% Pen-Strep), with or without a challenge of 100 ng/mL TNFα. There were three replicates per treatment group. The cells were incubated with the treatments for 48 hours at 37°C in 5% CO_2_. Following treatment, the cell layers were washed thrice with PBS, fixed in 4% (w/v) paraformaldehyde (Sigma Aldrich, St. Louis, MO) for 20 minutes at room temperature, and then washed twice in PBS. The cells were permeabilised for 60 minutes at room temperature in permeabilising solution (Triton X-100 0.2% (v/v), normal goat serum 1% (v/v) (Life Technologies, Carlsbad, CA), sodium azide 0.1% (w/v) in PBS). Cell layers were incubated at room temperature overnight with the primary antibodies, 1:250 dilution polyclonal rabbit anti-occludin and 1:500 dilution monoclonal mouse anti-ZO-1 (Life Technologies, Carlsbad, CA) in permeabilising solution, simultaneously. The cells were washed three times for 15 minutes in 0.1% (v/v) triton X-100 in PBS. Cells were incubated with secondary antibodies, 1:250 dilution anti-occludin Alexa 555 goat anti-rabbit IgG and 1:250 anti-ZO-1 Alexa 488 goat anti-mouse (Life Technologies, Carlsbad, CA) in PBS, for 2 hours at room temperature protected from light. Following incubation cells were washed five times for 5 minutes each using wash solution. Membranes were cut free from insert support using a scalpel and forceps and placed onto glass slides. Two drops of anti-fade mounting media (ProLong® Gold Antifade, Life Technologies, Carlsbad, CA) was applied per membrane, coverslips were added and sealed to prevent leaking, and then left overnight in the dark to cure. The slides were viewed using an Olympus Fluoview FV10i confocal microscope and images captured using the FV10-ASW (version 3.1b) software. The secondary antibodies dyes were selected from the list available within the software and images were taken under 60x magnification. For each membrane, three images of randomly selected areas were taken (total n = 9 images per treatment group). Exported TIFF images were analysed using the Coloc 2 plugin in Fiji (version ImageJ 1.51h) software [24] to determine the level of co-location between the two labelled proteins, ZO-1 and occludin. The red and green channels were separated and background subtraction was completed using a rolling ball radius set at 50.0 pixels. Threshold regression was set to Costes (25 Costes randomisations selected), whole image was analysed (ROI/mask was set to none), point spread function (PSF) was set to 3.0. The Pearson’s Correlation value between the two colour channels was recorded.

### Statistical analysis

For the TEER and mannitol flux assays, treatments were compared in GenStat v17.1 using the ‘Repeated measures analysis of variance’ tool. If the *treatment x time* effect was significant (P<0.05), the means of individual treatments at a given time point were compared using the least significant difference at 5% (LSD 5%). For the confocal microscopy, the Pearson’s Correlation values of the co-location of ZO-1 and occludin were transformed using Fishers Transformation to normalise the data. The values were compared in GenStat v17.1 using ANOVA with *treatment* as the factor, followed by pair-wise comparisons of treatments using Tukey’s 95% confidence intervals test.
